# Simple phantom fabrication for MRI‐based HDR brachytherapy applicator commissioning

**DOI:** 10.1002/acm2.13039

**Published:** 2020-10-05

**Authors:** Jessica M. Fagerstrom, Sukhjit Kaur

**Affiliations:** ^1^ Northwest Medical Physics Center Lynnwood WA USA; ^2^ Kaiser Permanente Seattle WA USA

**Keywords:** brachytherapy, HDR, image guidance, MRI

## Abstract

A new high dose rate (HDR) brachytherapy program was initiated in a community hospital setting, with the goal of using magnetic resonance (MR) images with the implant in place during the planning process. Physics acceptance testing and commissioning was completed for key program components, including multiple applicators. To image new applicators for MRI‐based planning prior to use with patients, agar gel doped with copper sulfate was created using simple, MR‐safe household materials as a practical and inexpensive alternative to custom‐machined precision phantoms. Applicators in‐phantom were scanned in a 1.5 T MRI scanner using the same sequences developed for the brachytherapy program, then rigidly registered to high‐resolution computed tomography (CT) images to assess distortion, artifact, and geometric displacement. To date, Varian tandem and ring sets, segmented cylinders, cervical probes, endometrial applicators; and third‐party plastic needles, tandems, and vaginal guides have been imaged in phantom and are available for use clinically.

## 1 INTRODUCTION

Currently, magnetic resonance imaging (MRI) represents the gold standard for target delineation in image‐guided high dose rate (HDR) brachytherapy.^1^ Implementing MRI in an HDR brachytherapy program depends on available resources, and three broad workflow categories of MRI's use in planning may be defined: MRI informed, MRI based, and MRI guided.^2^ Here, MRI‐informed brachytherapy describes the use of previously acquired MR images to inform optimal applicator placement at the time of implant. The MRI does not include the implant. MRI‐based brachytherapy describes the use of MRI for planning after the implant is in place, with planning either based solely on the MR images, or based on MRI that is registered to a computed tomography (CT) dataset. MRI‐guided brachytherapy describes the use of real‐time MR image guidance to place the brachytherapy implant intraoperatively. Based on available resources and because of the potential for substantial tissue distortion due to the implant itself, MRI‐based brachytherapy was considered preferable for this institution.

Careful commissioning of applicators is essential for safe and effective HDR treatment.^3^, ^4^, ^5^ Part of this process is three‐dimensional (3D) image acquisition. Previous work has described commissioning performed in preparation for MRI‐based and MRI‐guided HDR programs, including phantom measurements. This has been done using water phantoms: for example, Owrangi et al^6^ and Zhang et al.^7^ performed MRI measurements in water phantoms and described their implementation processes for incorporating MRI into their HDR brachytherapy programs. Zhang et al. noted that a gel phantom would have been preferable to water, but that the institution did not have a gel phantom available. Water‐based phantoms do offer a nontoxic, inexpensive, and readily available option; however, they require approximately 10 min of settling time and are susceptible to vibrations.^8^ For these reasons, water phantoms may be impractical when several commissioning scans are required at a clinical center that maintains a full MRI schedule. Haack et al.^9^ and Kim et al.^10^ describe precision‐machined acrylic phantoms designed to suspend needles, tandems, rings, and ovoid applicators in gel or a copper sulfate solution for MR imaging. These custom phantoms were developed and machined by those groups to include an internal stereotactic coordinate system. For this work, it was desired to fabricate simple, MR‐safe, inexpensive gel phantoms for scanning in both CT and MRI without the time delay or expense of designing and manufacturing precision‐machined custom acrylic phantoms. Although simple phantoms lack the internal stereotactic coordinate system of more advanced phantom designs, rigid registration with high‐resolution CT data provides a method to review rigid geometry between the imaging modalities.

## 2 MATERIALS AND METHODS

A Varian Bravos HDR afterloader (Varian Medical Systems, Inc., Palo Alto, CA) was purchased with several Varian applicators and third‐party components. Third‐party, single‐use components included 12‐ and 15‐cm vaginal guides (Alpha‐Omega Services, Inc., Bellflower, CA); plastic 16‐gauge, 30‐cm Flexi needles with friction cuffs and tungsten obturators (Best Medical International, Inc., Springfield, VA); and 32‐cm, 6‐mm‐diameter polymer intrauterine tandems (Liberty Medical, Inc., Sterling, VA). The polymer tandems are to be used with Varian 32‐cm, 1.8‐mm‐diameter mould probes, which are not present at the time of MRI. Other Varian reusable components included titanium universal endometrial applicator sets (similar to Rotte “Y” applicators), universal segmented cylinder applicator sets, universal cervix probe sets, 60° 3D interstitial ring and tandem applicator sets, 90° 3D interstitial ring and tandem applicator sets, and 45° ring and tandem applicator sets.

All Varian reusable parts imaged in the study were deemed by the manufacturer as MR conditionally approved (with all specified conditions met by the planned use), or MR safe. The Varian universal endometrial applicator set included nonferrous metallic components within the scanning region (titanium tandems), while other Varian applicator components used in the study were composed of the durable plastics polyetheretherketone (PEEK) and polyphenylsulfone (PPSU) along with titanium elements outside of the field of view to accommodate the ClickFit^TM^ connections to transfer guide tubes. Single‐use third‐party equipment included in the study were the vaginal guides composed of polyoxymethylene (Delrin®), disposable needles composed of plastic (with no metallic obturator in place for scanning), and intrauterine tandems composed of a proprietary polymer material. Prior to phantom fabrication, the institution’s MR safety committee reviewed and approved all components to be used for the program.

**Fig. 1:**
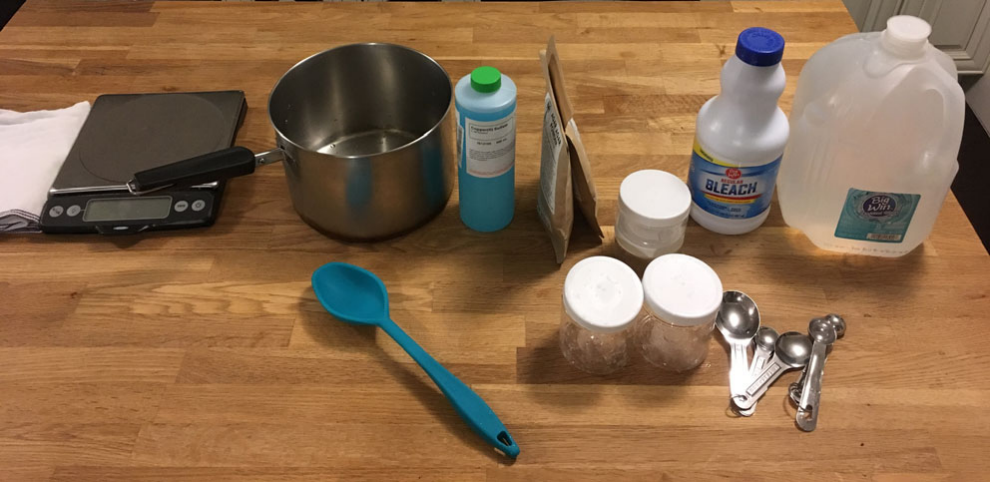
Simple supplies used for phantom construction. Dilute bleach was used to sterilize the surfaces of all components prior to fabrication, and a solution of agar‐agar powder, distilled water, and copper sulfate were used for the gel.

Inexpensive plastic storage containers (Sterilite Corporation, Townsend, MA) were obtained for the phantom bases, and a mold room electron block foam cutter was used to create custom foam inserts, cut to the shape of individual applicators and to fit snugly into the phantom bases. The foam inserts were affixed within the plastic containers, and were used to suspend the applicators within the central volume of the phantoms. Gels from agar, and agar's purified form, agarose, have been described in the literature for use in MRI phantoms for their tissue‐mimicking properties.^8^, ^11^ Additives such as gadolinium chloride or nickel chloride also may be used to adjust relaxation properties of gels.^8^ For this work, food grade agar‐agar powder (Landor Trading Co., Montreal, Canada) was used with distilled water and copper sulfate solution (Aldon Corporation, Avon, NY) for fabrication in a simple home kitchen. A photograph of supplies is included in Fig. 1.

**Fig. 2:**
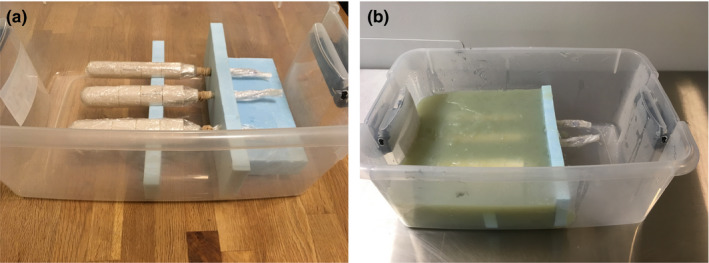
Three diameters of the Varian universal segmented cylinders are shown (a) wrapped in plastic wrap and set on custom‐cut foam blocks during phantom fabrication preparation, and (b) in the finished phantom.

Single‐use components, including the third‐party plastic needles, Delrin vaginal guides, and polymer intrauterine tandems, were set in phantoms directly. Multiple‐use Varian applicators were first wrapped in a thin layer of plastic wrap to separate them from the gel prior to positioning within the phantom bases. Due to the geometry of the applicators, the plastic wrap unavoidably included a small amount of air in the volume immediately surrounding the phantom, although care was taken to minimize this volume. Protective caps were used to assure no gel would be introduced into source channels. All utensils and the phantom bases were sterilized with a dilute bleach solution prior use. Based on the literature,^9^, ^11^ a gel mixture was created using ratios of 1‐L distilled water, 30‐g agar‐agar powder, and 5‐mL (approximately 1.0 teaspoon) 0.1 M CuSO_4_ solution. The agar‐agar powder and water were brought to a boil on a stove top over high heat, with constant stirring. Once boiling, the mixture was removed from the heat, and the CuSO_4_ solution was added. The gel was cooled to approximately 48°C, stirring occasionally, to achieve a pourable and homogeneous consistency that would be cool enough not to damage the applicators. Once sufficiently cool but before the gel had set, the warm gel was poured into the prepared phantom bases around the preset applicators and allowed to solidify completely. For phantoms including needles, the gel was poured and allowed to cool completely in the phantom base, and then needles were inserted into the solid phantoms. Needles inserted multiple times resulted in extraneous voids in the gel. These voids remained visible in imaging studies, so care should be taken to insert needles only once. Photographs of one of the phantoms are included in Fig. 2.

Completed phantoms were scanned in both CT and MRI using the protocols designed for gynecological HDR patient imaging. Computed tomography imaging was completed using a LightSpeed RT16 CT scanner (General Electric Healthcare, Milwaukee, WI) with a technique of 120‐kV, 97‐mA, 0.625‐mm slice thickness, and a field of view of 25.0 cm. MR imaging was completed using a 1.5‐T MAGNETOM Aera scanner (Siemens Healthcare, Erlangen, Germany) with body flex receiver coils. MR sequences included 3D, T2‐weighted (T2W) 1‐mm isotropic pixel size scans at 440‐Hz sequence readout bandwidth, with the center of the phantom volume positioned at the MRI scanner isocenter. Computed tomography and MR image data were loaded into the BrachyVision treatment planning system (Varian Medical Systems, Inc., Palo Alto, CA) v.15.5 for further analysis.

## 3 RESULTS AND DISCUSSION

The MR images were rigidly registered with the CT datasets, where the high‐resolution CT was considered the benchmark for geometric fidelity. Registration was accomplished within Eclipse software (v.15.5, Varian Medical Systems, Palo Alto, CA) first using pixel data with an automatic mutual information algorithm and a region of interest including the entire rigid container in both the MRI and CT scans. Manual registration tools were then used when needed to fine‐tune the relative image positions based on the applicators. Initial image registration was completed by the lead brachytherapy certified medical dosimetrist and reviewed by the physics team via slice‐by‐slice verification. As noted by Hellebust et al.,^3^ it is crucial that image registration efforts concentrate on aligning the position of the applicators between datasets (as opposed to, for example, aligning bony structures, in the case of patient data, or rigid phantom materials, in the case of phantom scans). Artifact and distortion in MRI are dependent on a large range of factors, including field strength, array coils, pulse sequences, applicators, and gradient distortion corrections, which is why imaging applicators in phantom is recommended prior to their use in patients. For this work, geometric displacement of applicators between rigidly registered CT and MR images was found to be <1 mm for all combinations of MR and CT images.

**Fig. 3:**
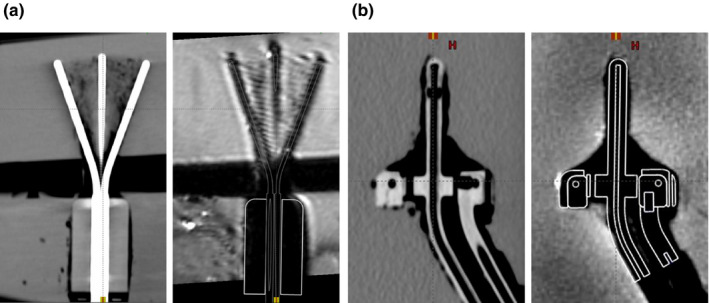
Components of the Varian universal endometrial applicator set (a), and the Varian three‐dimensional interstitial ring applicator set (b), are shown imaged in gel. Computed tomography (CT) images are shown on the left and magnetic resonance (MR) images are shown on the right with the vendor‐provided digital models overlaid. For the endometrial applicator set, the straight central tandem, left 20 × 70 mm tandem, and right 20 × 70 mm tandem with the vaginal cylinder were included in the displayed region of the phantom. For the ring set, the 30 × 36 mm 60° ring probe and the 40‐mm‐intrauterine tandem were included in the displayed region of the phantom. Air gaps introduced by using protective plastic wrap around reusable applicator components are visible in both CT and MR images.

Figure 3 includes registered images for one of the configurations of the universal endometrial applicator set and one of the configurations of a 3D interstitial tandem and ring set. In the MR images, the applicators appear as a black void in contrast to the gel. In CT images, it was possible to visualize the source channel directly based on the contrast between the air in the source channel and the applicator material for all tested components except the titanium tandems from the universal endometrial sets. Vendor‐provided digital models were available for all reusable Varian components imaged in this study, including the endometrial applicator set tandems. No models were available for third‐party components. Vendor‐provided digital models, where available, were reviewed on both CT and MR images and satisfactory geometric agreement was found. AAPM's forthcoming TG‐236 report is expected to address the use of model libraries in the context of intracavitary brachytherapy. Currently, these applicators will be used within a workflow that includes both MRI for target delineation and CT for applicator reconstruction and organ‐at‐risk segmentation. With these needs in mind, the rigid registration of the datasets was considered sufficient.

A practical item to note regarding the timing between phantom construction and scanning is that agar gel has a limited shelf life once prepared. Literature suggests including toxic additives to the gel such as sodium azide may be helpful for slowing mold formation (although such additives may affect MR relaxation characteristics),^8^ but for this study, shelf life was considered adequate after disinfecting phantom components and surfaces prior to fabrication and keeping the phantoms covered when not in use. Furthermore, some applicators may have a specified maximum period of use (e.g., the Varian instructions for use documentation for the universal segmented cylinder set indicates that the applicators are intended for use for less than 30 days of contact with patients). Therefore, it is recommended to minimize the overall time between phantom fabrication, scanning, and final retrieval of applicators from within the phantom. Decreasing the time between CT and MRI scanning will also serve to minimize the likelihood of inadvertently disturbing the position of the applicators within the phantom between scans.

When using plastic wrap to protect reusable applicator components, air will be introduced between the applicator and the plastic wrap and is expected to be visible in phantom. This is noticeable in Fig. 3. If possible, further inhomogeneities in the phantoms should be avoided by heating a large enough batch of gel to fill the phantom container to the desired capacity, then pouring the gel in a single action. Some amount of susceptibility artifact in MR images is anticipated because the magnetic susceptibility of applicators is expected to differ from that of tissue and agar gel, especially in the case of the endometrial applicator set in which a (nonferrous) metal‐tissue interface is present. Air introduced in the phantom gel medium by bubbles in the gel and gaps between protective plastic wrap and the applicator will be visible in both CT and MR images, while susceptibility artifacts will be visible in MR images only. A key aim of the phantom studies is to verify that the MR imaging that will be acquired with the applicators in place, including expected susceptibility artifacts, is satisfactory for the needs of the planned use. For this institution, this process was used to verify that the planned MRI sequences could be rigidly registered to the high‐resolution CT dataset.

A recent survey of clinical practice patterns of medical physicists and dosimetrists subscribing to international email listservers indicated that only approximately 3% of respondents use MRI for verification of brachytherapy applicator positioning.^12^ With MRI offering superior soft tissue contrast and better demarcation of target(s) compared to CT, this population is expected to increase. The AAPM’s upcoming task group on MRI guidance in HDR brachytherapy (TG‐303, in development) will offer clinical physicists recommendations on many aspects of HDR programs incorporating MRI, including commissioning. In general, 3D imaging is appropriate for the commissioning of new applicators, and phantoms using sand, uncooked rice, or other similar materials for suspending applicators will not generate signal in MRI. Water phantoms offer a simple and inexpensive alternative, but with the required settling time and vibrational effects, may be unrealistic at clinics that do not have many available MRI appointment times for additional physics scans. Agar gel phantoms offer a practical option. While previous publications detail precision‐machined phantoms with agarose or CuSO_4_ solution, simple rapidly prototyped agar gel phantoms may be used for clinics intending to use CT for applicator digitization and MRI for target delineation.

## 4 CONCLUSION

This work summarizes phantom fabrication from inexpensive MR‐safe materials that were readily commercially available, or were already on hand in a standard clinical radiotherapy block room. The phantoms themselves were constructed in a simple home kitchen with basic tools, using ratios of gel components of 1‐L distilled water, 30‐g agar‐agar powder, and 5‐mL 0.1 M CuSO_4_ solution (approximately 1 teaspoon). MR‐conditionally approved applicators were scanned in fabricated phantoms in both CT and MRI, using the MR sequences developed for gynecological HDR patient planning. The scan data were rigidly registered in the treatment planning system software, and the applicators were approved for clinical use. At the time of submission, segmented cylinders, cervical probes, endometrial applicators, rings, tandems, vaginal guides, and plastic needles have all been imaged prior to clinical use.

## CONFLICT OF INTEREST

There is no relevant conflict of interest to disclose.
